# Ricin Toxin Activates the NALP3 Inflammasome

**DOI:** 10.3390/toxins2061500

**Published:** 2010-06-17

**Authors:** Meghan Lindauer, John Wong, Bruce Magun

**Affiliations:** Department of Cell and Developmental Biology, Oregon Health and Science University, 3181 S.W. Sam Jackson Park Rd., Portland, OR 97239, USA; Email: lindauem@ohsu.edu (M.L.); wongjo@ohsu.edu (J.W.)

**Keywords:** ricin, NALP3, inflammasome, IL-1

## Abstract

Ricin exhibits well characterized ribotoxic actions that lead to the inhibition of protein synthesis and the phosphorylation of stress activated protein kinases (SAPKs). Proinflammatory effects of ricin are thought to be caused by upregulation of genes encoding proinflammatory transcripts as a result of the activation of c-Jun *N*-terminal kinase (JNK) and p38 MAPK. We reported previously that macrophages and interleukin-1β (IL-1β) signaling are required for murine host immune responses to ricin delivered to the lungs. Here we report that ricin-mediated IL-1β release from bone-marrow derived macrophages is dependent on the NALP3 inflammasome, a scaffolding complex that mediates pro-IL-1β cleavage to active IL-1β by caspase-1. Release of IL-1β from macrophages was suppressed by the reactive oxygen species (ROS) scavenger *N*-acetyl cysteine (NAC) and high extracellular K^+^, which are two agents known to inhibit NALP3/cryopyrin/CIAS1 inflammasome formation. By employing inhibitors of p38 MAPK and JNK, we demonstrated that ricin-mediated release of IL-1β was enhanced, rather than suppressed, by inhibition of SAPK phosphorylation. In contrast, proteasomal inhibitors bortezomib and MG-132 completely suppressed ricin-induced IL-1β release from macrophages. These data suggest that ricin-mediated translational inhibition itself, by fostering the disappearance of labile protein(s) that normally suppress inflammasome formation, may constitute the mechanism underlying IL-1-dependent inflammatory signaling by ricin.

## 1. Introduction

Ricin is a potent inhibitor of protein synthesis that triggers a robust proinflammatory response when administered to mice and non-human primates [1]. Mounting evidence points to the macrophage as the specific cell type responsible for ricin’s lethal effects *in vivo* [2,3,4]. In primary bone-marrow derived- (BMDM) and alveolar macrophages, exposure to ricin causes the phosphorylation of stress activated protein kinases (SAPKs) p38 MAPK and JNK, and triggers the transcriptional upregulation of genes encoding proinflammatory cytokines and chemokines [3,5]. *In vivo*, depletion of macrophages prior to delivery of ricin to the pulmonary system of mice results in diminished inflammatory signs, including reduced neutrophilia and pulmonary edema [6]. 

IL-1β signaling is an essential component of ricin-mediated inflammation. Mice deficient in IL-1α/β or IL-1R display enhanced survival as well as decreased neutrophilia and pulmonary edema compared to wild-type mice after exposure to ricin [6]. Ricin’s ability to induce neutrophilia can be restored in IL-1α/β-deficient animals by the co-administration of exogenous IL-1β with ricin. Furthermore, the IL-1 receptor antagonist anakinra (Kineret®) provides protection against ricin-mediated inflammatory effects in wild-type mice, confirming a central role of IL-1 in ricin toxicity.

IL-1β secretion by macrophages is a tightly regulated process involving at least two distinct signals [7,8]. Macrophages first require priming by Toll-like receptor (TLR) ligands (such as LPS) or by cytokines (such as TNFα or IL-1β itself) in order to induce the expression of pro-IL-1β. The pro-form of IL-1β is inactive and requires cleavage by the cysteine protease caspase-1 for maturation and secretion [9]. Molecular platforms called inflammasomes stimulate caspase-1 activity and are necessary for IL-1β processing [10,11]. One of the best-characterized inflammasome complexes is the NALP3 (or NLRP3/cyropyrin) inflammasome. This multiprotein complex includes the NOD-like receptor (NLR) family member NALP3, the cysteine protease caspase-1, and the adaptor protein apoptosis-associated specklike protein (ASC), which facilitates interaction of caspase-1 with NALP3 [12,13].

NLRs such as NALP3 are intracellular sensors of pathogen-associated molecular patterns (PAMPs) and danger-associated molecular patterns (DAMPs) [14]. Unlike AIM2, an NLR family member that senses dsRNA through direct ligand binding, NALP3 is thought to be a more general sensor of cellular stress through a mechanism that is not fully understood [15]. NALP3 inflammasomes are activated by a diverse array of agents including exogenous danger signals such as bacterial RNA, *Candida albicans*, and influenza [16,17,18], environmental stressors such as silica and asbestos [19,20,21], and endogenous danger signals such as ATP, uric acid and amyloid β [22,23,24]. 

While a singular mechanism cannot yet explain the activation of the NALP3 inflammasome by its wide array of activators, two events that may be common to all known activators are the generation of reactive oxygen species (ROS) and the efflux of potassium [20,21,25,26]. ROS generation by NALP3 agonists is believed to indirectly cause activation of the inflammasome via the ROS-sensitive TXNIP protein [27]. Studies show that inhibitors of ROS can reduce the amount of mature IL-1β released by cells after stimulation with ATP, asbestos, and silica [21,28]. However, work on human monocytes lacking functional NADPH oxidase did not show a connection between ROS and NALP3 inflammasome activation [29], suggesting that ROS generation may not always be required. In regards to the role of potassium, it is thought that the intracellular assembly of NALP3 inflammasomes requires a low potassium environment [30]. Activation of caspase-1 is suppressed by normal levels of intracellular potassium [31], and two laboratories have shown that the efflux of K^+^ can directly promote the oligomerization of ASC and its association with caspase-1 [26,32]. 

Here we report that ricin toxin is an activator of the NALP3 inflammasome. We found that ricin-mediated IL-1β release from primary bone marrow-derived macrophages not only required the expression of NALP3, ASC and caspase-1, but also was inhibited by co-treatment of cells with the ROS scavenger *N*-acetyl-cysteine (NAC) or high extracellular potassium. In addition, we found that the activation of JNK and p38 MAPK by ricin was not inhibited by either NAC or elevated extracellular potassium, suggesting that ricin mediates IL-1β release from cells in a manner that is independent of its ability to activate kinases. 

Proinflammatory consequences of ricin are thought to be initiated by phosphorylation of ZAK, a MAP3K that transduces the signal through downstream kinases p38 MAPK and JNK and leads to proinflammatory gene expression [33]. We found that treatment of cells with inhibitors reported to bind to ZAK [34,35] (Nilotinib and Sorafenib) led to inhibition of p38 and JNK activity but failed to block ricin-mediated release of IL-1β from macrophages. We hypothesized that inhibition of protein synthesis might be the event that triggers activation of the NALP3 inflammasome by contributing to the disappearance of important repressor proteins from the cell. By employing proteasome inhibitors to suppress proteasomal degradation of cellular proteins, we found that ricin-induced IL-1β release from macrophages required active proteasomes. Together these data describe a novel mechanism governing ricin-mediated inflammatory signaling.

## 2. Materials and Methods

### 2.1. Reagents and Antibodies

Ricin was purchased from Vector Laboratories (Burlingame, CA). LPS (L-2630), *N*-acetyl cysteine (NAC), and bovine-pancreas insulin (I6634) were purchased from Sigma-Aldrich (St. Louis, MO). Trichloroacetic acid (TCA) was purchased from Fisher Scientific (Pittsburgh, PA). Nilotinib, Sorafenib and Bortezomib were obtained from LC Laboratories (Woburn, MA). MG-132, the p38-specific inhibitor SB203580, and the JNK inhibitor SP600125 were purchased from EMD Biosciences (Gibbstown, NJ). The mouse IL-1β enzyme-linked immunosorbent assay (ELISA) Ready-Set-Go was purchased from eBioscience (San Diego, CA). Anti-IL-1β was purchased from Abcam (Cambridge, MA), anti-ASC was purchased from Enzo Life Sciences (Lausen, Switzerland), and anti-p38, anti-cryopyrin (H-66)/NALP3, and anti-caspase-1 (M-20) were purchased from Santa Cruz Biotechnology (Santa Cruz, CA). Anti-phospho-SAPK/JNK (9251S), anti-phospho-p38 MAPK (9211S), anti-phospho-MAPKAP-2 (3041S) were purchased from Cell Signaling Technologies (Danvers, MA). 

### 2.2. Animals and Animal Procedures

All animal procedures were performed according to protocols approved by the Institutional Animal Care and Use Committee at Oregon Health and Science University, Portland, Oregon. C57BL/6J and Caspase-1 deficient mice were purchased from The Jackson Laboratory (Bar Harbor, ME). ASC-deficient and NALP3-deficient mice were kindly provided by V. Dixit (Genentech, San Francisco, CA). Male mice, 8–10 weeks of age, were used throughout the experiments. Before experimental procedures, mice were anesthetized intraperitoneally with 80 mg/kg of ketamine and 10 mg/kg of xylazine. 

### 2.3. Isolation and Treatment of Bone Marrow-Derived Macrophages

Bone marrow-derived macrophages were prepared from C57BL/6J, ASC^−^^/^^−^, Caspase-1^−^^/^^−^, and NALP3^−^^/^^−^ mice. Marrow was flushed from femurs and tibias with PBS and cultured in α-Minimum Essential Medium (α-MEM, Cellgro, Herndon, VA), supplied with 10% Fetal Bovine Serum (FBS, Cellgro, Herndon, VA), 50 μg/mL gentamicin, and 100 ng/mL recombinant mouse Colony Stimulating Factor 1 (CSF-1, R&D Systems, Minneapolis, MN) for 72 h on non-tissue culture treated 10-cm petri dishes. Cells were passaged and cultured for an additional 72h. Cells from one confluent 10-cm dish were plated into one 6-well tissue culture plate (Sarstedt, Newton, NC) for an additional 24 h before experiments. Cells were serum-deprived in α-MEM for 30 min followed by treatment with 50 ng/mL LPS for 4 h. Cells were rinsed with fresh media and exposed to 0.01 µg/mL ricin in serum-free media for an additional 4 h prior to harvesting. In experiments involving co-treatments with ricin plus *N*-acetyl-cysteine (30 mM), high potassium (130 mM), Nilotinib (1 µM), Sorafenib (1 µM), SB203580 (10 µM), SP600125 (20 µM), bortezomib (0.5 μM) or MG-132 (10 μM), cells were exposed to indicated concentrations of inhibitors. In experiments involving high potassium, potassium was substituted for sodium in the medium in order to maintain equivalent ion concentrations. 

### 2.4. Immunoblotting and IL-1β ELISA

BMDM cells were lysed in 2X ESB lysis buffer in preparation for immunoblotting. Equal volumes of the cell lysates were separated on a 10% denaturing polyacrylamide gel in the presence of sodium dodecyl sulfate and were transferred onto polyvinylidene difluoride membranes according to standard laboratory procedures. Proteins from BMDM media supernatants were precipitated using TCA and run on 13% gels. Briefly, samples were incubated at 4 °C overnight with 200 µg insulin carrier and 400 µL ice-cold 100% TCA prior to centrifugation at 12,000 rpm for 5 min. Pellets were air dried and resuspended in 2X ESB lysis buffer followed by separation on a 13% denaturing polyacrylamide gel. Membranes were incubated with the indicated antibodies and the corresponding horseradish peroxidase-conjugated secondary antibodies; signals were detected using enhanced chemiluminescence. Media supernatants were analyzed in triplicate using IL-1β ELISA (eBioscience) according to the manufacturer’s protocol.

### 2.5. Statistical Analysis

Individual groups were compared using unpaired *t* test analysis and were interpreted in a two-tailed manner.

## 3. Results

### 3.1. Ricin Stimulates IL-1β Release from Wild-Type Bone Marrow Derived Macrophages

**Figure 1:**
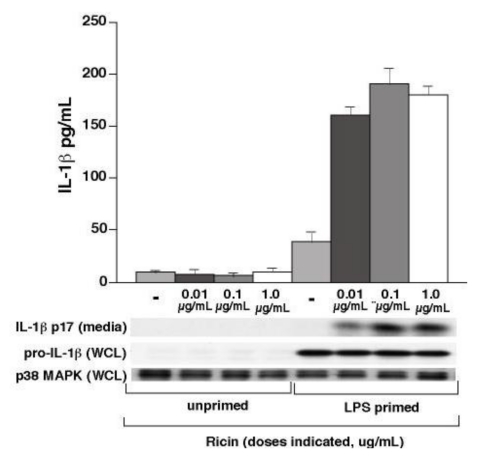
Ricin stimulates IL-1β release from WT BMDM. Secreted IL-1β was measured by ELISA analysis of media supernatants collected from LPS-primed WT BMDM after 4 h ricin exposure. Bars represent the mean ± s.d. of triplicate wells. Results are representative of three individual experiments. Media supernatants were also precipitated and subjected to immunoblot analysis for detection of IL-1β p17. WCLs were subjected to immunoblot analysis for detection of pro-IL-1β and p38 MAPK as a loading control.

Studies *in vivo* and *in vitro* have demonstrated that macrophages constitute primary targets of ricin [3,5,6]. Wild-type (WT) alveolar and bone-marrow derived macrophages respond similarly to ricin in that they both display phosphorylation of p38 MAPK and JNK in dose-dependent manner inversely proportional to the ricin-mediated decrease in levels of protein translation [3]. In order to determine if macrophages release IL-1β in response to these same doses, wild-type BMDM were first primed for 4 h with LPS (50 ng/mL) to induce pro-IL-1β expression, after which cells were rinsed and exposed to varying concentrations of ricin for 4 h (Figure 1). Whole cell lysates (WCL) and media supernatants were subjected to immunoblotting and ELISA for detection of IL-1β. Ricin alone did not induce expression of pro-IL-1β. Priming with LPS led to an increase in levels of 37 kDa pro-IL-1β in cell lysates, and subsequent exposure to ricin led to the appearance of processed 17 kDa IL-1β in the media. Processed IL-1β was detected in media supernatants for all doses tested. We chose to use the lowest dose, 0.01 µg/mL, for all subsequent experiments. The B subunit of ricin is responsible for binding to cell surfaces, but lacks the *N*-glycosidase activity of the A subunit that is responsible for depurination of 28S rRNA. Exposure of LPS-primed macrophages to purified ricin B subunit failed to elicit appearance of p17 IL-1β in the culture medium (data not shown), suggesting that interactions between ricin holotoxin and surface molecules do not elicit signals that elicit processing of pro-IL-1β.

### 3.2. Ricin-Mediated Release of IL-1β Requires NALP3, ASC and Caspase-1

**Figure 2:**
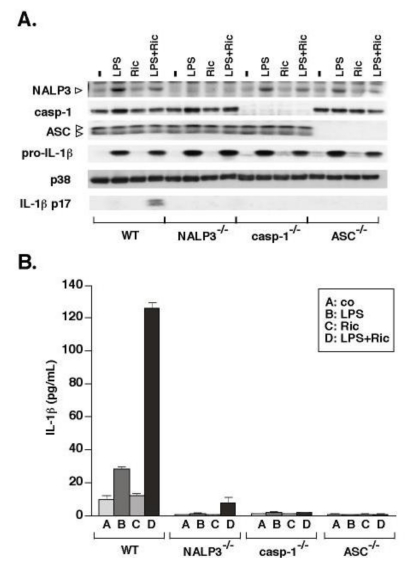
Ricin-mediated release of IL-1β requires NALP3, ASC, and caspase-1. Primed and unprimed WT, NALP3^−^^/^^−^, casp-1^−^^/^^−^, and ASC^−^^/^^−^ BMDM were treated with ± 0.01 µg/mL ricin or vehicle alone (co) for 4 h. WCLs were subjected to immunoblotting for NALP3 inflammasome components, pro-IL-1β and p38 MAPK. Media supernatants were either precipitated for immunoblot analysis (A) or subjected to ELISA for measurement of secreted IL-1β (B). Bars represent the mean ± s.d. of triplicate wells.

Secretion of processed IL-1β from cells requires discrete events, including the accumulation of pro-IL-1β through a priming step and the assembly of the inflammasome, a multiprotein scaffolding complex that both activates caspase-1 and brings caspase-1 into close proximity with pro-IL-1β [7,10]). The best-studied inflammasome complex is the NALP3 inflammasome, in which NALP3 associates with caspase-1 through the adaptor protein ASC [12,13]. The NALP3 inflammasome has been shown to mediate cellular responses to several danger-associated molecular patterns: exogenous danger signals such as silica and asbestos [19,21], and endogenous danger signals such as ATP and uric acid [22,23,36]. To determine if macrophages sense and respond to ricin through the NALP3 inflammasome, we compared the ricin-mediated responses of WT cells to responses of cells deficient in NALP3, ASC, or caspase-1 (Figure 2). BMDM from each mouse strain were primed with LPS for 4 h prior to ricin treatment. After 4 h exposure to ricin, cells were harvested and processed for immunoblotting (Figure 2A) to examine expression levels of inflammasome components and pro-IL-1β. Media supernatants were subjected to detection of IL-1β by immunoblotting and ELISA (Figure 2B). Although pro-IL-1β was similarly induced in the LPS-primed cells of each strain, the processing of pro-IL-1β to active IL-1β was significantly decreased in cells deficient in either NALP3, ASC or caspase-1. These results suggest that the NALP3 inflammasome is required for ricin-mediated IL-1β processing and release from BMDM.

### 3.3. Elevated Extracellular K^+^ and *N*-Acetyl-Cysteine Prevent Ricin-Mediated Secretion of IL-1β

**Figure 3:**
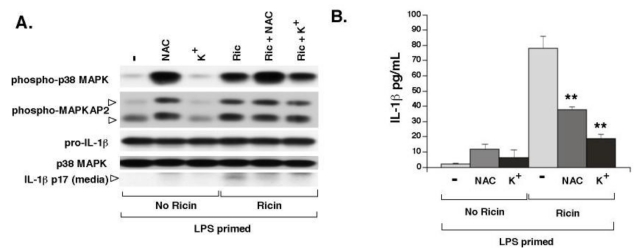
Elevated extracellular K^+^ and NAC prevent ricin-mediated secretion of IL-1β from WT BMDM. Primed WT cells were treated ± 0.01 µg/mL ricin in combination with either NAC or K^+^ for 4 h. A) WCLs were subjected to immunoblotting for phospho-p38 MAPK, phospho-MAPKAP2, pro-IL-1β and p38 MAPK as a loading control. Media supernatants were either precipitated for immunoblotting (A) or subjected to ELISA for determination of secreted IL-1β (B). Bars represent the mean ± s.d. of triplicate wells (**: p < 0.01).

If NALP3 mediates the release of IL-1β from macrophages exposed to ricin, then agents shown to inhibit the activity of the NALP3 inflammasome should block the appearance of IL-1β in the medium after exposure to ricin. To address this question we employed two inhibitors of the NALP3 inflammasome: increased extracellular potassium (K^+^) and the ROS scavenger, *N*-acetyl-cysteine. High extracellular K^+^ blocks IL-1β release caused by a variety of danger signals that activate NALP3 including asbestos, silica, and ATP [37]. *In vitro* studies of inflammasome activation suggest that NALP3 inflammasome assembly requires a low K^+^ intracellular environment [26]. In addition, activation of NALP3 is reportedly blocked by ROS inhibitors like NAC through a mechanism that is not well understood [25]. Wild-type BMDM were primed with LPS for 4 h, after which cells were co-treated with increased extracellular potassium or NAC and ricin. Four hours later, cells and media supernatants were harvested and processed for immunoblot analysis and ELISA. Media collected from cells co-treated with ricin and either NAC or elevated extracellular K^+^ contained 50% and 75% less IL-1β respectively, than cells treated with ricin alone. Untreated cells and cell exposed to NAC or elevated K^+^ expressed equivalent amounts of pro-IL-1β (Figure 3). Interestingly, exposure of cells to NAC or elevated K^+^ did not diminish ricin-mediated phosphorylation of p38 MAPK or the p38 MAPK target, MAPKAP2. Furthermore, NAC by itself led to phosphorylation of p38 MAPK and MAPKAP2 while diminishing the release of IL-1β from ricin-treated cells, suggesting that ricin-mediated inflammasome activation and SAPK phosphorylation are not necessarily linked. 

### 3.4. Ricin-Mediated Phosphorylation of p38 MAPK and JNK Is Not Required for Ricin-Mediated IL-1β Secretion

**Figure 4:**
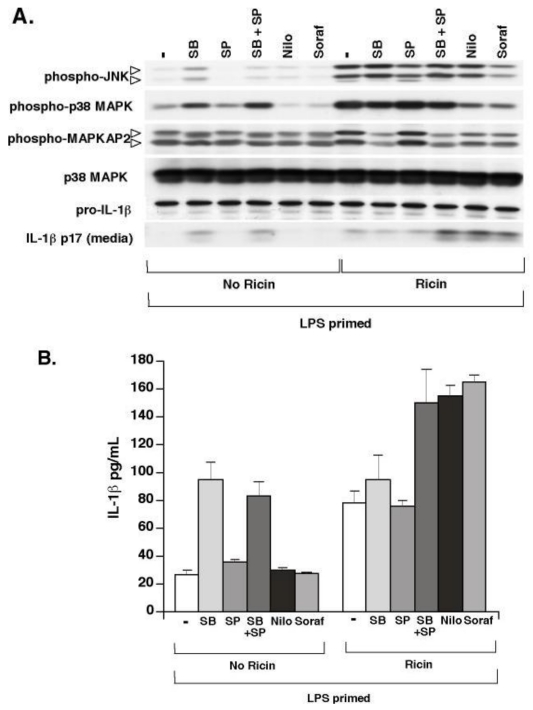
Ricin-mediated phosphorylation of p38 MAPK and JNK is not required for ricin-mediated IL-1β secretion. Primed WT cells were treated ± 0.01 µg/mL ricin in combination with either SB203580, SP600125, SB + SP, Nilotinib or Sorafenib for 4 h. (A) WCLs were subjected to immunoblotting for phospho-JNK, phospho-p38 MAPK, phospho-MAPKAP2, pro-IL-1β and p38 MAPK as a loading control. Media supernatants were either precipitated and subjected to immunoblotting (A) or analyzed for IL-1β by ELISA; (B) Bars represent the mean ± s.d. of triplicate wells.

If ricin-mediated phosphorylation of SAPKs is an upstream event leading to NALP3 activation, then blocking the phosphorylation of these kinases should prevent the appearance of IL-1β in the media of ricin-treated cells. To address this question we employed SB203580, an inhibitor of p38 MAPK, and SP600125, an inhibitor of JNK. We also employed two inhibitors (Nilotinib and Sorafenib) that have been reported to have very high affinity for the ATP-binding site of ZAK [34,35], the upstream MAP3K that is phosphorylated by ricin and other ribotoxic stressors [33]. LPS-primed cells were treated with SB203580 (SB), SP600125 (SP), Sorafenib, Nilotinib or ricin, alone and in combination for 4 h, at which time cell lysates and media supernatants were collected (Figure 4). Phosphorylated SAPKs and pro-IL-1β levels were examined in cell lysates by immunoblotting. Processed IL-1β from media supernatants was detected using both immunoblotting and ELISA. Inhibitors exhibited varying degrees of effectiveness in suppressing SAPKs. JNK activation was decreased marginally by SP and significantly by Sorafenib; p38 MAPK activation was diminished significantly by Nilotinib and Sorafenib; and the activation of MAPKAP2, a target of p38 MAPK, was significantly decreased by SB, Nilotinib, and Sorafenib. Surprisingly, SB stimulated IL-1β release from macrophages on its own, and cells co-treated with kinase inhibitors and ricin secreted similar or greater amounts of IL-1β as cells treated only with ricin, despite diminished levels of phosphorylated p38 MAPK and JNK in these cells. These data suggest that SAPK activation by ricin is not a requirement for the release of IL-1β from ricin-treated cells and that suppression of ricin-mediated ZAK activation did not reduce ricin-induced activation of the NALP3 inflammasome.

### 3.5. Proteasome Inhibitors Block Ricin-Mediated Release of IL-1β from WT BMDM

**Figure 5:**
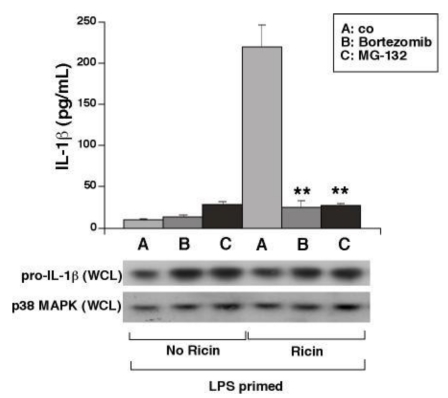
Proteasome inhibitors block ricin-mediated release of IL-1β from WT BMDM. Primed cells were treated ±0.01 µg/mL ricin and either bortezomib, MG-132, or vehicle alone (co) for 4 h. Secreted IL-1β was measured by ELISA from media supernatants. Bars represent the mean ± s.d. of triplicate wells (**: p < 0.01). WCLs were subjected to immunoblotting for detection of pro-IL-1β and p38 MAPK as a loading control.

Importantly, the dose of ricin used in this study (0.01 µg/mL) elicits a 50% inhibition of protein synthesis by 3 h in addition to its effects on SAPKs and the release of IL-1β [3]. We hypothesized that inhibition of protein synthesis per se could lead to activation of NALP3 by contributing to the disappearance of labile proteins which may regulate the availability of NALP3 to participate in formation of the inflammasome. In such case, proteasomal activity may be required for ricin-induced activation of NALP3 and release of IL-1β from macrophages. To test this hypothesis, we employed the specific and potent proteasome inhibitors bortezomib and MG-132. Although cells exposed to bortezomib or MG-132 expressed similar levels of pro-IL-1β to cells exposed only to LPS or ricin, cells exposed to bortezomib or MG-132 exhibited complete suppression of ricin-mediated secretion of IL-1β (Figure 5). These data are consistent with the notion that translational inhibition itself may be a mechanism by which ricin triggers IL-1β dependent inflammatory signaling.

## 4. Discussion

Previously we demonstrated that, in addition to inhibiting protein synthesis, ricin triggers the phosphorylation of JNK and p38 MAPK. Activation of these kinases is required for ricin-mediated expression of mRNAs encoding inflammatory cytokines and chemokines [38,39]. Furthermore, we showed that ricin administration to the lungs results in a neutrophilic inflammation *in vivo* that is dependent on IL-1β and on the presence of macrophages [6]. Experiments in BMDM and alveolar macrophages demonstrated that macrophages constitute primary targets for ricin, and are a likely source of ricin-induced production of IL-1β [3,5,6]. However, the mechanism of ricin-mediated processing and release of IL-1β was unclear. 

Here we report that ricin triggers IL-1β release from macrophages in a NALP3-, ASC- and caspase-1-dependent manner, and that ricin-mediated IL-1β release, but not ricin-mediated SAPK phosphorylation, may be blocked by treatment of cells with high extracellular potassium or the ROS scavenger, NAC. In addition, we found that IL-1β release is not dependent upon p38 or JNK activation, since inhibitors that target the p38 MAPK and JNK pathways failed to block IL-1β release and indeed, even enhanced IL-1β secretion from primed cells. Furthermore, treatment of macrophages with proteasome inhibitors in combination with ricin demonstrated that ricin-mediated IL-1β release was dependent upon the proteasomal degradation of cellular proteins. Taken together, these data suggest a novel mechanism for ricin-mediated inflammatory signaling, and helps to explain why inhibitors of IL-1 signaling are capable of diminishing ricin’s inflammatory effects.

The major finding of this study is that macrophages require components of the NALP3 inflammasome (NALP3, ASC, and caspase-1) in order to process and release active IL-1β after ricin exposure (Figure 2). NALP3 is a Nod-like receptor protein containing a pyrin-domain (PYD) through which it interacts with the PYD-containing adaptor protein, ASC, upon oligomerization [40]. ASC binds caspase-1 through its caspase-1 recruitment domain (CARD) and stimulates its activity, so that the IL-1β precursor may be cleaved into its active form, p17 IL-1β, and secreted by the cell in response to danger signals. Although primed WT BMDM expressed similar levels of pro-IL-1β as the NALP3-, ASC-, and caspase-1-deficient BMDM (Figure 2a), the mutant BMDM all failed to release IL-1β into the medium after ricin treatment. These results suggest that the NALP3 inflammasome is required for ricin-mediated processing of IL-1β. 

As a sensor of a myriad of microbial and non-microbial danger signals and the culprit of pyrin-associated autoinflammatory diseases caused by mutations in the *nlrp3* gene [41,42], the NALP3 inflammasome is well-studied but still poorly understood. Unlike TLRs, which bind directly to their ligands [43], studies suggest that NALP3 may instead sense an intermediate molecule produced by its activators. While the list of activators grows longer each year, the mechanism through which dissimilar signals trigger NALP3 activity remains unclear. 

Although insufficient to activate the NALP3 inflammasome on their own, potassium efflux and ROS production are common events that occur during NALP3 activation by all known activators [25]. Evidence suggests that ASC oligomerization and caspase-1 activation require a low potassium environment [26,32]. Indeed, high extracellular potassium applied to macrophages to prevent ricin-mediated potassium efflux resulted in significantly diminished IL-1β secretion in ricin-treated cells (Figure 3), despite expressing normal levels of pro-IL-1β after priming. Furthermore, the ROS-scavenger NAC was able to reduce IL-1β release by 50% in ricin-treated BMDM (Figure 3), consistent with the notion that intracellular ROS may be permissive for activation of the NALP3 inflammasome.

In order to determine whether SAPK phosphorylation was required for ricin-mediated inflammasome activation, we employed inhibitors known to have high affinity for ZAK (Nilotinib and Sorafenib) as well as inhibitors specific to the p38 MAPK (SB203580) and JNK (SP600125). Surprisingly, we found that inhibiting ricin-mediated SAPK activation enhanced, rather than suppressed, IL-1β release from primed macrophages (Figure 4). These data suggest that SAPK phosphorylation by ricin may suppress the NALP3 inflammasome rather than contribute to its activation. The partial suppression of ZAK by kinase inhibitors failed to diminish NALP3 activation, suggesting that NALP3 activation by ricin occurs by a mechanism that does not involve ZAK. However, the possibility that ZAK contributes to ricin-mediated activation of the NALP3 inflammasome cannot be excluded at this time.

Another possibility is that ricin induces activity of the NALP3 inflammasome through the inhibition of cellular protein translation per se by depleting cells of proteins that suppress formation of inflammasome complexes under normal conditions. The existence of a family of small proteins that have emerged as important inflammasome regulators lends merit to this idea. These proteins contain either pyrin-only domains (POPs) or CARD-only domains (COPs) and act as endogenous dominant-negative modulators of inflammasome activity [44]. One might imagine a scenario in which their disappearance from the cell could trigger activation of the inflammasome as a result of ricin-induced inhibition of protein synthesis combined with normal protein turnover. 

To explore this possibility, we treated WT BMDM with two different proteasome inhibitors, bortezomib and MG-132, and examined ricin-mediated IL-1β release under conditions in which proteasomal degradation of cellular proteins was blocked. Our experiments demonstrated that both bortezomib and MG-132 signficantly reduced IL-1β secretion from WT cells exposed to ricin, supporting the hypothesis that ricin-mediated translational inhibition itself may lead to activation of the NALP3 inflammasome by fostering the disappearance of labile protein(s). It would be interesting to examine whether other inhibitors of protein translation similarly activate inflammatory signaling through NALP3. 

Since host immune responses triggered by toxins are nonproductive and often deleterious, investigation into mechanisms underlying inflammatory signaling by toxins is warranted so that we might better understand and manage toxin-induced pathologies.

## 5. Conclusions

When added to LPS-primed murine bone marrow-derived macrophages, ricin induces the processing of pro-IL-1β to IL-1β, which is secreted into the culture medium. The conversion ofpro-IL-1β to IL-1β is prevented in macrophages deficient in NALP3, ASC, or caspase-1, suggesting that ricin induces the formation of the NALP3 inflammasome. The mechanism by which ricin mediates the secretion of IL-1β through formation of the NALP3 inflammasome does not involve the stimulation of JNK and p38 MAPK or activation of ZAK. Proteasome inhibitors suppress ricin-mediated inflammasome activation, suggesting that the turnover of labile suppressor protein(s) may be responsible for restraining the formation of the NALP3 inflammasome.
